# The role of HIV Tat protein in HIV-related cardiovascular diseases

**DOI:** 10.1186/s12967-018-1500-0

**Published:** 2018-05-08

**Authors:** Yanan Jiang, Lu Chai, Moyondafoluwa Blessing Fasae, Yunlong Bai

**Affiliations:** 1 Department of Pharmacology (State-Province Key Laboratories of Biomedicine- Pharmaceutics of China, Key Laboratory of Cardiovascular Research, Ministry of Education), College of Pharmacy, Harbin Medical University, Harbin, China; 2 Translational Medicine Research and Cooperation Center of Northern China, Heilongjiang Academy of Medical Sciences, Harbin, China

**Keywords:** HIV, Tat, Cardiovascular diseases

## Abstract

The human immunodeficiency virus (HIV) is a major global public health issue. HIV-related cardiovascular disease remains a leading cause of morbidity and mortality in HIV positive patients. HIV Tat is a regulatory protein encoded by *tat* gene of HIV-1, which not only promotes the transcription of HIV, but it is also involved in the pathogenesis of HIV-related complications. This review is aimed at summarizing the current understanding of Tat in HIV-related cardiovascular diseases.

## Background

The human immunodeficiency virus (HIV) is a major global public health issue. It has claimed more than 35 million lives so far. In 2016, 1 million people died from HIV-related causes globally [1]. With the advent of highly active antiretroviral therapy (HAART), the life expectancy of HIV patients was extensively increased and HIV infection has become a chronic disease [2, 3]. Presently, alleviation of the complications induced by long term HIV infection remains an unresolved problem. Among all HIV-related complications, cardiovascular disease remains a leading cause of mortality in HIV positive patients [4]. Cardiovascular involvement in HIV-infection was first described in 1983 by Autran et al. who noted myocardial Kaposi’s sarcoma at autopsy [5]. Thereafter, additional research revealed the association between HIV infection and cardiovascular diseases. HIV infection increased the risk of cardiovascular disease, which is independent of antiretroviral therapies [6, 7]. Though some antiretroviral drugs, such as zidovudine, have a deleterious effect on myocardium, HAART has dramatically decreased the incidence of cardiomyopathy and mortality of HIV infected patients [8, 9]. Therefore, over the past few decades, scientific research has increasingly focused on unraveling the mechanism and role of HIV-infection in the pathogenesis of cardiovascular diseases

## The characteristics of HIV Tat

The RNA genome of HIV consists of at least nine genes, including *gag*, *pol*, *env*, *tat*, *rev*, *nef*, *vif*, *vpr*, and *vpu*. Tat stands for “trans-activator of transcription”, which is a small nuclear protein encoded by the *tat* gene in HIV-1. It consists of 86–101 amino acids depending on the subtype. During the process of HIV replication, Tat serves as a trans-activator that drastically enhances the efficiency of viral transcription [10, 11]. Tat is encoded by two exons, highly conserved regions (1–72 amino acids) and sub-type dependent regions (73–86 to 101 amino acids). The first region contains five functional domains which are: the acidic/proline rich, the cysteine-rich/ZnF, the core, the basic and the glutamine-rich domains. The first three regions constitute a minimal activation domain. The basic region contains nuclear localization signal and RNA-binding domain. The cysteine-rich/ZnF region represents the protein transduction domain [12]. The structure of Tat gives it the properties of both transcription promotion and membrane transduction.

## HIV Tat and cardiovascular diseases

### HIV Tat and cardiomyopathy

HIV infection is recognized as an important cause of dilated cardiomyopathy. Cohen et al. reported the connection between HIV infection and dilated cardiomyopathy for the first time in 1986 [13]. HIV-related cardiomyopathy with reduced systolic function occurred much more frequently before the advent of HAART therapy or in developing countries in which HAART is not widely available [8, 14, 15]. After HAART with excellent virologic control, most patients are minimally symptomatic or have diastolic dysfunction [8, 16–18].

HIV-transgenic mice (Tg26) is a well-described murine line of NL4-3 Δ*gag/pol* that expresses HIV-related proteins and develops HIV-related complications in the hemizygotes [19]. Under basal conditions, WT and Tg26 mice have normal systolic and diastolic function. Cardiac contractile dysfunction in the isolated work-performing heart from Tg26 mice was originally reported by Lewis et al. which was worsened by antiretroviral treatment with zidovudine [20]. Similarly, Cheung et al. found that Tg26 mice exhibits normal cardiac contractile function under basal conditions, but with less tolerance on surgical stress and the down regulation of Bcl2-associated athanogene 3 (BAG3) induced contractile abnormalities [21]. Though they observed different phenotype in HIV transgenic mice, there were similarities that transgenic expression of HIV facilitates cardiac dysfunction.

Further study proved that Tat protein plays an important role in HIV-related cardiac dysfunction. Targeted myocardial HIV Tat transgenic mice showed a depression in myocardial systolic and diastolic functions [22]. The occurrence of cardiac dysfunction was proved to be related with mitochondrial ultrastructural damage. Profound cardiac mitochondrial damage has been associated with the death of homozygote +/+ Tat mice within 14 days, in contrast to +/− Tat high and +/− Tat low mice which developed, matured and survived up to 2 years [23].

Latest research reveals that Tat has profound impacts on cardiomyocytes. Transfection of adenoviral-Tat impaired the uptake of mitochondrial Ca^2+^ ([Ca^2+^]_m_) and the electrophysiological activity of cardiomyocytes, which also aggravated hypoxia/re-oxygenation-induced cardiomyocyte injury through interference with the initiation of autophagy and the clearance of autophagic proteins [24]. It is worth mentioning that methamphetamine, a cardiotoxic stimulant, is abused epidemically and is frequently associated with acquisition of HIV-1 infection.

A study addressed the individual and combined effects of HIV-1 and methamphetamine on cardiac dysfunction using a transgenic mouse model of HIV infection. The results demonstrated that Tat and methamphetamine increased calcium uptake and promoted mitochondrial dysfunction in cardiomyocytes, this altered the DNA methylation and expression of *CACNA1C*, that encodes the alpha 1c (Cav1.2) subunit of the L-type calcium channel [25].

### HIV and arrhythmia

Corrected QT (QTc) prolongation is predictive of cardiovascular mortality. Frequent occurrence of QT interval prolongation have been found in HIV-positive patients [26, 27]. The application of HIV protease inhibitors (PIs) are suspected to be responsible for HIV-related long QT syndrome (LQTs) through the blockade of cardiac rapidly activating delayed rectifier K^+^ current (I_Kr_) channels encoded by human ether-a-go-go-related gene (*hERG*) [28]. A study proved that a low CD4+ cell count is associated with LQTs, specifically in this study, the HAART was not associated with QTc prolongation in HIV-positive patients [29].

In HIV transgenic mice, cardiac repolarization is prolonged compared with wild-type littermates, which is at least partially attributed to the reduction of transient outward K^+^ current (I_to_). Thus, it appears that HIV itself contributes to the delayed repolarization observed in the transgenic mice [30]. Subsequent studies explored the effect and the underlying mechanisms of Tat in cardiomyocytes. It was also observed that Tat protein prolonged QTc interval in guinea pig. Correspondingly, isolated cardiomyocytes form Tat-treated guinea pig exhibited prolonged action potential duration at 90% of repolarization (APD_90_) and reduced I_Kr_ [31]. Furthermore, incubation of Tat protein also significantly decreased I_Kr_ in hERG stably expressed HEK293 cells through the inhibition of hERG protein expression but with no effect on the hERG mRNA expression [31]. Similar results were achieved in human induced pluripotent stem cell derived cardiomyocytes (hiPSC-CMs). Tat transfection in heterologous expression systems led to a decrease in I_Kr_ and I_Ks_ through sequestering membrane phosphatidylinositol-(4,5)-bisphosphate (PIP_2_), with no significant alteration in I_Ca-L_ [32].

In addition, Tat could affect both cardiomyocytes and neurons thus inducing bradycardia. Transgenic mice with myocardial overexpression of Tat exhibits a relative bradycardia phenotype, which is associated with reduced susceptibility of adrenergic responsiveness [22]. Further study proposes that Tat also excites cardiac parasympathetic neurons of nucleus ambiguus and produces a sustained bradycardia. Tat-induced Ca^2+^ elevation in neurons was largely attributed to the promotion of lysosomal Ca^2+^ mobilization, Ca^2+^ release via inositol 1,4,5-trisphosphate-sensitive endoplasmic reticulum pools, and Ca^2+^ influx via transient receptor potential vanilloid type 2 (TRPV2) channels [33].

### HIV Tat and atherosclerosis

HIV-seropositive patients are at higher risk for atherosclerosis than HIV-seronegative persons. This has been variably attributed to antiretroviral treatment, advanced immunodeficiency, and HIV-associated inflammation [34, 35]. Priscilla Y et al. proposed that increased atherosclerosis with HIV infection occurs in the absence of antiretroviral therapy, detectable viremia, or overt immunodeficiency [36]. Francisci D et al. also proposed that HIV infection induces alterations of markers of endothelial function, which is independent of antiretroviral treatment [37]. Therefore, persistent HIV-associated inflammation and endothelial dysfunction might be potential causes for accelerated atherosclerosis in HIV infection.

Carotid intima-media thickness (IMT), an indicator of generalized atherosclerosis, is higher in HIV patients than in age-matched control subjects, which is associated with classic coronary risk factors and with low CD4+ T cell count [38]. Beyond traditional cardiovascular disease risk factors, low CD4+ T-cell count is the most robust risk factor for increased subclinical carotid atherosclerosis in HIV-infected patients [39]. In animal models, HIV transgenic mice exhibit endothelial dysfunction with increased intima-media thickness and arterial stiffness. Furthermore, the arteries from HIV transgenic mice show decreased elastin content, increased cathepsin K and cathepsin S activity and mechanical residual stress. Thus, HIV transgenic mice exhibition of pre-clinical markers of vascular remodeling are consistent with early on-set atherosclerosis [40]. Although there is no direct evidence that Tat causes atherosclerosis, circumstantial evidence implicates a relationship between them. Tat significantly decreased endothelium-dependent vasorelaxation and endothelial nitric oxide synthase (eNOS) production in porcine coronary arteries [41].

### The effect of Tat on inflammation and immune activation

Chronic immune activation and inflammation are risk factors of cardiovascular diseases. HIV-infection is also associated with increase in inflammation factors and immune activation. The inflammatory and coagulation biomarkers IL-6, hsCRP and D-dimer expression levels are associated with an increased risk of cardiovascular disease in HIV-infected individuals [42]. The immune hyperactivation of HIV-infected T cells is mediated by Tat [43]. Low concentrations (0.1–1 nM) of Tat protein increase the anti-apoptotic ability of CD4+ T lymphoblastoid Jurkat cells through stimulation of the catalytic activity of phosphatidylinositol 3-kinase (PI3K)/Akt pathway [44]. Although HAART suppresses HIV replication, it is often unable to restore immune homeostasis [45]. Thus, immunization with Tat acts in synergy with HAART to help in restoring immune homeostasis [46].

The inflammatory vascular environment is critical for the initiation and development of atherosclerosis. Tat induces vascular inflammation through the activation of nuclear factor-kappa B (NF-κB) in human vascular endothelial cells (HUVECs) and leads to the upregulation of inflammatory mediators, including IL-1β, MCP-1, VCAM-1 and E-selectin, which could be attenuated by estrogen [47]. Further study found that Tat stimulated upregulation of intercellular cell adhesion molecules specifically ICAM-1 in HUVECs was through the suppression of miR-221/-222 in a NF-κB-dependent pathway [48]. Tat-related impairment of the survival and differentiation of mesenchymal stem cells might play an important role in vessel damage and formation of the atherosclerotic lesions observed in HIV-infected patients and this could be considered an additional important mechanism involved in promoting vascular damage and atherosclerosis in the course of HIV disease [49]. In addition, Tat could mediate the induction of adhesion molecules and also function as an exogenous cytokine in the activation of human endothelial cells, which upregulates E-selectin expression, enhances the secretion of IL-6, and synergizes with TNF in mediating these effects [50].

### HIV Tat and pulmonary arterial hypertension

HIV infected patients have a greater incidence of pulmonary arterial hypertension (PAH) compared to the general population. Specifically, in comparison with the incidence of idiopathic PAH in the general population (1–2 per million), HIV-infected patients have a 2500-fold increased risk of developing PAH [51]. The development of HIV-related PAH reduces the probability of survival by half as compared with HIV-infected individuals without HIV-related PAH [52]. Depending on gender and ethnic differences, the proportion of mortality due to pulmonary heart disease and diseases of pulmonary circulation in HIV-infected adults ranges from 4.3% to 17.5% of overall cardiovascular disease mortality in a cohort of HIV-infected adults in the United States [53]. The advent of HAART ameliorates the outcome of patients with HIV-related PAH [52, 54].

HIV infection and intravenous drug abuse have been identified as common independent risk factors of PAH. For example, morphine potentiates HIV Tat protein-mediated apoptosis and proliferation of pulmonary endothelial cells, which may have led to strikingly more pronounced pulmonary vascular remodeling than exposed alone [55]. The processes of PAH progression include the transformation of endothelial cells from initial apoptosis to apoptosis-resistant hyper-proliferation. A recent study found a potential link between autophagy and HIV-mediated enhancement of proliferation of apoptosis-resistant pulmonary endothelial cells. The initial exposure of pulmonary endothelial cells to morphine and Tat results in independent activation of both autophagy and apoptosis. However, chronic increase in autophagy reduces the incidence of oxidative stress and apoptosis that allows the cells to adapt to stress leading to cell survival and uncontrolled proliferation of pulmonary endothelial cells. Therefore, morphine in combination with Tat results in the induction of autophagy in pulmonary endothelial cells that may lead to an increase in severity of angio-proliferative remodeling of the pulmonary vasculature [56].

## Tat transgenic mice

Transgenic technology has made it possible to investigate the pathogenic mechanism of HIV in mice. HIV transgenic mice develops HIV-infection related complications. Some HIV encoded proteins, including Tat, Nef and Vpr, are important determinants for the pathogenicity of HIV [57, 58]. Tat is one of the extensively investigated proteins, which is sufficient to induce some HIV-related complications in the absence of HIV infection. To clarify the specific role of Tat, a model of transgenic mice expressing Tat protein was established. Tat expression induces dermal lesions resembling Kaposi’s sarcoma and facilitates the generation and progression of tumors/cancers of different histotypes in transgenic mice [59–62]. In Tat-transgenic mice, Tat expression is sufficient to cause neuropathologies similar to most of those noted in the brain of HIV-infected patients [63]. Tat is an immunological manipulator, which increases the production of pro-inflammatory cytokines and induces lymphoid hyperplasia [64, 65]. With an α-myosin heavy chain promoter, Tat was selectively expressed in mice cardiac myocyte. Targeted myocardial transgenic expression of HIV Tat in mice results in relative bradycardia and contractile dysfunction, this model has helped to provide a better understanding of the pathophysiological mechanisms of Tat in HIV-related cardiomyopathy [22, 23].

## Conclusions

**Fig. 1:**
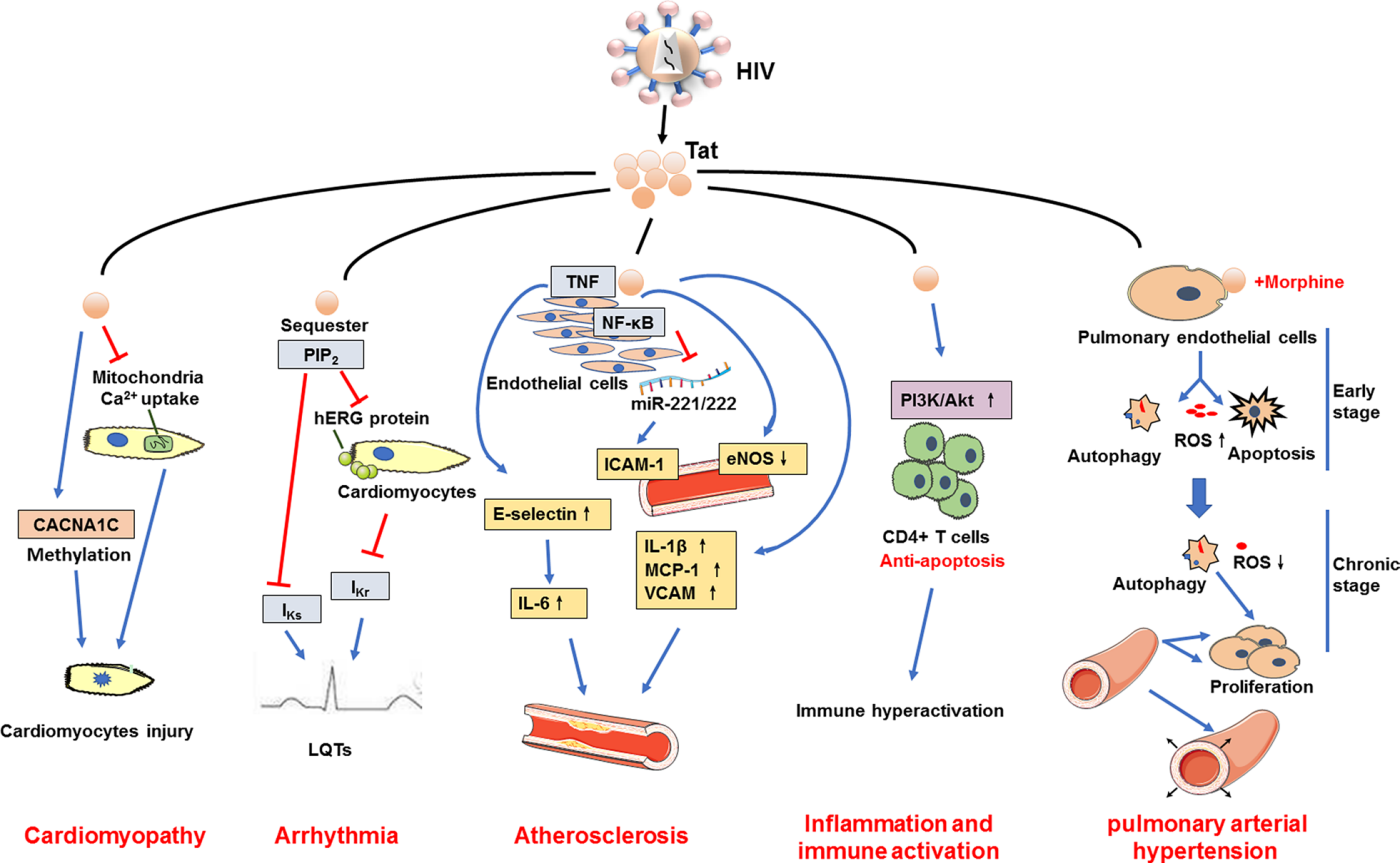
HIV Tat and HIV-related cardiovascular diseases

All these findings confirmed that Tat protein not only promotes the transcription of HIV but also participates in the pathogenesis of HIV-associated cardiovascular complications (Fig. 1). It is a particularly good target for prevention, treatment of HIV and development of antiviral drugs. Tat-specific antibodies appear to be an excellent method to prevent HIV acquisition and spreading. It has been proven to prevent and/or control infection with pathogenic simian/human immunodeficiency virus (SHIV) in nonhuman primates [66, 67].

In recent years, a randomized phase II exploratory clinical trial found that Tat immunization is safe, well tolerated and could induce anti-Tat antibodies in most patients. It promoted a durable and significant restoration of immune homeostasis and induced a significant reduction of blood proviral DNA in patients on effective combination anti-retroviral therapy regimen. Thus, Tat immunization represents a promising pathogenesis-driven intervention to intensify HAART efficacy [68]. HIV-1 Oyi was a strain cloned from a seropositive patient [69]. Tat Oyi vaccine can generate neutralizing antibodies against Tat variants regardless of their mutations, thereby establishing a rationale for testing Tat Oyi as a potential therapeutic vaccine in human trials [70]. The Tat Oyi vaccine in association with combination anti-retroviral therapy may provide an efficient means of controlling the HIV-infected cell reservoir [71]. Furthermore, as a cell penetrating peptide, Tat has been developed as a drug delivery system in the treatment of cancer [72]. However, Tat and anti-cancer drugs both have cardiovascular toxicity. Whether they have synergistic injury effect on cardiovascular system remains unknown.

Besides the conventional therapy involved in circumventing the effect of TAT in HIV- related cardiovascular diseases, there are also natural herbal extracts reported to possess anti-HIV properties. For instance, Curcumin is a natural herb extract, which has anti-HIV activity. A recent study revealed that curcumin causes inhibition and degradation of Tat which may be one of the major mechanisms behind its anti-HIV activity [73]. Also, Curcumin has been shown to possess anti-inflammatory and ameliorative properties, thereby playing a protective role in cardiovascular disease [74]. These findings suggest that harnessing the potential efficacy of natural herbs might be a source of new drugs for the treatment of HIV infection and related cardiovascular complications, however further studies could still be carried out on Curcumin in relation to HIV-related cardiovascular diseases.

This review has been able to show the effects of Tat on HIV-related cardiovascular diseases. Further investigation is still needed to elucidate the molecular mechanism of Tat and HIV-related cardiovascular disorders which may suggest novel insights and therapeutic targets.

## Abbreviations

Akt: protein kinase B
APD_90_: action potential duration at 90% of repolarization
BAG3: Bcl2-associated athanogene 3
[Ca^2+^]_m_: mitochondrial Ca^2+^
eNOS: endothelial nitric oxide synthase
HAART: highly active antiretroviral therapy
hERG: human ether-a-go-go-related gene
hiPSC-CMs: human induced pluripotent stem cell derived cardiomyocytes
HIV: human immunodeficiency virus
HUVECs: human vascular endothelial cells
I_Kr_: rapidly activating delayed rectifier potassium current
I_to_: outward potassium current
LQTs: long QT syndrome
NF-κB: nuclear factor-kappa B
PIs: Protease inhibitors
PIP_2_: phosphatidylinositol-(4,5)-bisphosphate
PI3K: phosphatidylinositol 3-kinase
QTc: corrected QT
Tat: trans-activator of transcription
TRPV2: transient receptor potential vanilloid type 2
